# Effects of tillage and maturity stage on the yield, nutritive composition, and silage fermentation quality of whole-crop wheat

**DOI:** 10.3389/fpls.2024.1357442

**Published:** 2024-03-27

**Authors:** Liuxing Xu, Guojian Tang, Dan Wu, Yan Han, Jianguo Zhang

**Affiliations:** ^1^Department of Grassland Science, South China Agricultural University, Guangzhou, China; ^2^College of Agronomy and Life Sciences, Zhaotong University, Zhaotong, China; ^3^School of Biological Sciences and Technology, Liupanshui Normal University, Liupanshui, China

**Keywords:** maturity, nutritive value, whole-crop wheat, no-tillage, yield

## Abstract

Whole-crop wheat (*Triticum aestivum*, WCW) has a high nutritional value and digestibility. No-tillage (NT) can reduces energy and labor inputs in the agricultural production process, thus decreasing production costs. There are many studies on planting techniques of WCW at present, few being on no-tillage planting. This study aimed to compare the effects of different tillage methods and maturity stages on the yield, nutritive value, and silage fermentation quality of WCW. The experiment included two tillage methods (NT; conventional tillage, CT), two maturity stages (flowering stage; milk stage), and three years (2016-2017; 2017-2018; 2018-2019). Years had a strong influence on the yield and nutritional composition of WCW. This was mainly related to the amount of rainfall, as it affects the seedling emergence rate of wheat. Although tillage methods showed no significant effects on the yield, plant height, and stem number per plant of WCW (*P* > 0.05), compared to CT, the dry matter (DM) and crude protein (CP) yields of NT decreased by 0.74 t/ha and 0.13 t/ha. Tillage methods showed no significant effects on the nutritive composition of WCW (*P* > 0.05). The WCW at the milk stage had greater DM (5.25 t/ha) and CP (0.60 t/ha) yields than that at the flowering stage (3.19 t/ha and 0.39 t/ha) (*P*< 0.05). The acid detergent fiber concentration of WCW decreased by 34.5% from the flowering to the milk stage, whereas water-soluble carbohydrates concentration increased by 50.6%. The CP concentration at the milk stage was lower than that at the flowering stage (*P*< 0.05). The lactic acid concentration of NT (17.1 g/kg DM) silage was lower than that of CT (26.6 g/kg DM) silage (*P*< 0.05). The WCW silage at the milk stage had a lower NH_3_-N concentration (125 g/kg TN) than that at the flowering stage (169 g/kg TN) (*P*< 0.05). Wheat sown by NT and CT was of similar yield and nutritional value, irrespective of harvest stages. WCW harvested at the milk stage had greater yield and better nutritional composition and silage fermentation quality than that at the flowering stage. Based upon the results of the membership function analysis, no-tillage sowing of wheat was feasible and harvesting at milk stage was recommended.

## 1 Introduction

China’s population accounts for 17% of the world’s population, and with its growth, the acceleration of urbanization process and the increase of income, food consumption patterns have gradually changed. From 1961 to 2017, the food consumption structure (grain and livestock products) of domestic population changed substantially in China. In the structure of daily food consumption, the proportion of grain had decreased (Ren et al., 2017). Nationally, the yield of livestock products (pork, beef and mutton) has increased from 4.83 × 10^7^ t in 2000 to 6.52 × 10^7^ t in 2017 (Xu et al., 2023). However, it is worth noting that the dairy intake of domestic residents is still insufficient due to scarce supply of forage (Lin et al., 2021). In this context, the Chinese government has formulated several policies to support forage production and processing. For example, to prevent competition with food crops for land, increasing the planting area of forage crops and exploiting new forage resources by using idle land resources (such as fallow lands) have effectively alleviated forage shortage. In general, winter fallow fields were planted with green manure (the objective of improving soil fertility through the cultivation of green crops was to increase the yield of subsequent crops) or fallow in southern China. In recent decades, fluctuations in geopolitics, international exchange rates, and energy prices (Alam et al., 2022), have led to rising feed costs, and livestock producers have had to consider additional ways to obtain more feed and protein.

In Australia’s high rainfall zone, dual-purpose wheat (*Triticum aestivum*) is used to increase meat and wool production (McGrath et al., 2021). In Brazil, dual-purpose wheat is used for grazing, which increases meat production and improves the profitability of integrated crop-livestock systems (Timm et al., 2020). The whole crop wheat (WCW) has a high water-soluble carbohydrate (WSC) concentration, which facilitates the production of high-quality silage (especially during the milk stage) (Filya, 2003). At the same time, WCW after ensilaged has a high lactic acid concentration, which makes it a high-quality silage (Xie et al., 2012). Regarding animal production performance, WCW harvested at dough-stage has a high starch concentration, which is completely digested in dairy cows (Randby et al., 2019). Additionally, some studies showed that feeding WCW silage to ewe increases their efficiency of energy utilization compared to grass silage (Higgins et al., 2020). In pursuit of higher dry matter (DM) and crude protein (CP) yields and better silage fermentation quality, WCW is typically harvested at the late milk or early dough stage (Xie et al., 2012). However, at more mature stages of WCW growth, widespread lignification of stem internodes and leaf sheaths occurs, which reduces the digestibility of forage straw (Weinberg et al., 1991). Therefore, despite lower yield, some producers may harvest WCW at the flowering stage (Xie et al., 2012).

Traditionally, early rice (*Oryza sativa*, planting in Apr.)-late rice (planting in Jul.)-fallow rotation has been a popular cropping practice in southern China. Winter fallow (from Nov. to Mar. the next year) is usually used to save materials, seeds, labor, and energy inputs, but it wastes water and land resources. More importantly, planting forage crops during the fallow period increases economic profits (Xu et al., 2021a). In addition, the utilization of winter green manure crops is also a traditional method for improving the grain yield of subsequent crops in southern China. However, this will lead to the loss of forage. In the previous study, we found that winter fallow fields can be used to plant a season of forage wheat (Xu et al., 2021b). In the light of the above background, the southern China region is currently planting winter wheat to increase the local supply of forage.

No-tillage (NT) can reduce soil erosion and increase soil nutrient concentration availability, hence is widely adopted in agricultural production. It also reduces energy and labor inputs in the agricultural production process, thus decreasing production costs. However, the effect of NT on grain yield remains inconsistent (Pittelkow et al., 2015). As an important component of the whole plant, the grain inevitably affects the yield and nutrition of WCW. There are many studies on planting techniques of WCW at present, few being on no-tillage planting. This study offers more options for feed producers, including: 1) compare the effects of different years and tillage methods on the yield, nutritive composition, and silage fermentation quality of WCW; 2) choosing maturity stage for harvesting WCW. We hypothesize that 1) NT improves WCW yield, nutritional value, and silage fermentation quality; 2) the yield and nutritional value of WCW at milk stage were higher than at the flowering stage.

## 2 Materials and methods

### 2.1 Site description

**Figure 1:**
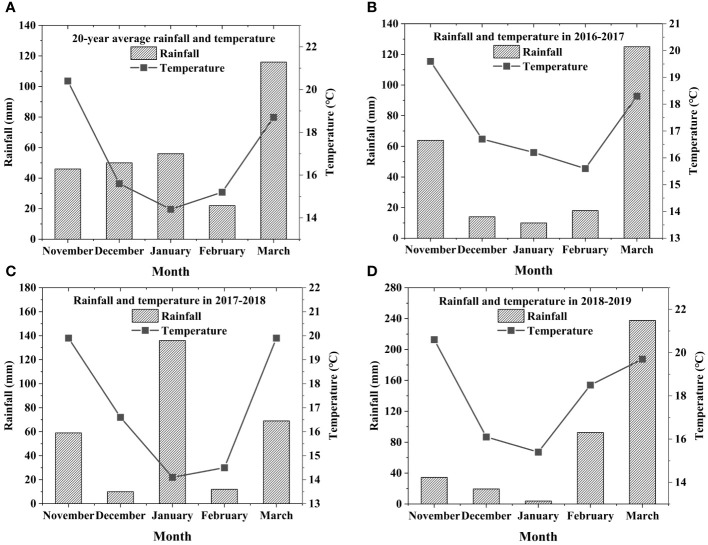
**(A)**, 20-year average rainfall and temperature; **(B)**, rainfall and temperature in 2016-2017; **(C)**, rainfall and temperature in 2017-2018; **(D)**, rainfall and temperature in 2018-2019.

The experiment was conducted at the experimental field of South China Agricultural University (23˚14′N and 113˚38′E), Guangzhou, Guangdong Province, China. The experiment location was a typical site of a Chinese region of rice and fallow (or green manure) cropping systems. Soil chemical properties (0-20 cm) were organic matter of 20.4 g/kg, total nitrogen of 0.82 g/kg, nitrate nitrogen of 102 mg/kg, available phosphorus of 12.1 mg/kg, and pH of 5.33 (three years mean values). The total rainfall during the winter crop growing periods in 2016-2017, 2017-2018 and 2018-2019 were 231, 286, and 388 mm, respectively (**Figure 1**). The average air temperature during the winter crop growing periods in 2016-2017, 2017-2018, and 2018-2019 were 17.3, 17.0, and 18.1°C, respectively (**Figure 1**).

### 2.2 Experimental design and management

The field trials were carried out from Nov. 2016 to Mar. 2017, Nov. 2017 to Mar. 2018 and Nov. 2018 to Mar. 2019. The experiment included tillage methods of NT and conventional tillage (CT) and maturity stages of the flowering stage and milk stage. Late rice was harvested in Nov. and then wheat was manually sown. For NT, the seeds were directly sown onto the soil surface, without tillage or action to improve the soil density. For CT, the soil was plowed with a pneumatic row direct drilling machine equipped with double disc furrow openers (XG788ZK, Guangzhou Agricultural Machinery Equipment, Guangzhou, China), and wheat seeds were manually scattered on the ground. Each plot was 3 m × 4 m, with an interval of 0.5 m between plots, and each treatment was in triplicate (randomly allocated). The wheat (Shimai-1) was sown at 285 kg/ha (NT and CT). No insecticides and fungicides were used during the experiments. WCW was not irrigated during the winter crop growing periods. The compound fertilizer (N: P_2_O_5_: K_2_O=15:6:8) was split-applied at 150 kg/ha. Forty percent was applied as a basal fertilizer, and the remaining fertilizer was applied at the jointing stage. The phenology of wheat was monitored according to the decimal code for the growth stages of cereals (Zadoks et al., 1974). **Table 1** shows the detailed field management and growth period of wheat. To fully utilize soil water, the wheat in 2016-2017 were planted on the third day after the harvest of late rice. However, in 2017-2018 and 2018-2019, the wheat was planted on the ninth and eleventh day after the harvest of late rice, when the soil water content was relatively low. Compared to 2016-2017, the emergence time of wheat in 2017-2018 and 2018-2019 was delayed by 5 and 8 days, respectively. In addition, due to differences in soil management methods, there were differences in the utilization of soil moisture between NT and CT, resulting in a lower seedling emergence rate for NT compared to CT (**Table 1**).

**Table 1:** Agricultural operations dates and Decimal Code (DC).

Years	Sowing date	Fertilization date		Harvesting date		Crop growth time (d)		Growth period		Population density (10^6^ plant/ha)	
		Base fertilizer date	Jointing fertilizer date	Flowering stage	Milk stage	Flowering stage	Milk stage	Flowering stage	Milk stage	No-tillage	Conventional tillage
2016-2017	11 Nov. 2016	24 Dec. 2016	22 Jan. 2017	22 Feb. 2017	14 Mar. 2017	104	124	DC69	DC77	2.24	3.06
2017-2018	22 Nov. 2017	28 Dec. 2017	25 Jan. 2018	25 Feb. 2018	20 Mar. 2018	96	119	DC66	DC75	1.28	1.80
2018-2019	23 Nov. 2018	3 Jan. 2019	21 Jan. 2019	23 Feb. 2019	19 Mar. 2019	93	117	DC65	DC75	2.14	2.89

Note: Decimal code.

### 2.3 Field sampling and silage making

The WCW was harvested when plants were at the flowering stage (DC65-69), and milk stage (DC 75-77, Zadoks et al., 1974). The fresh matter yield of WCW was determined with a 1 m^2^ (1.0 m × 1.0 m) quadrat, and the DM yield was calculated based on DM concentration and fresh matter yield. At harvest, 5.0 kg of fresh material was randomly selected (5 cm from the ground, mowed with a sickle) from each plot for the analyses of the number of epiphytic microbes, chemical composition, and silage making. Fresh materials were choped to 20 to 30 mm length, and mixed thoroughly after transporting to the laboratory. Then 300 g of materials were stuffed into bags (polyethylene plastic, 300 mm × 400 mm × 0.2 mm, Mingkang Packing Co. Ltd., Zhongshan, China), which were degassed and sealed using a vacuum sealing machine (Sinbo Vacuum Sealer, Hong Tai Home Electrical Appliance Co., Ltd., Hong Kong, China). All the bags were put in a laboratory cabinet (18 to 29°C, dark environment) for 60 days.

### 2.4 Microbial and chemical analyses

Lactic acid bacteria were cultured under anaerobic conditions at 37°C for 2 d (de Man-Rogosa-sharp agar), aerobic bacteria (nutrient agar), yeasts and molds (potato dextrose agar) were cultured under aerobic conditions at 37°C for 3 d. The DM concentration was measured at 70°C for 48 hours in an oven with forced air circulation (Blast drying oven 9003A, Shandong Jinan Haineng Instrument Co., Ltd., China Co., Ltd). CP and NH_3_-N concentrations were determined by Kjeldahl nitrogen determination (Nitrogen analyzer KN680, Shandong Jinan Alva Instrument Co., Ltd., Jinan, China) and ultraviolet spectrophotometer (Brodeick and Kang, 1980), respectively. The analyses of ether extract and crude fiber concentrations were conducted as outlined in AOAC (1990). Crude ash concentration was measured by burning in a muffle furnace at 550°C for 5 hours. Crude fiber (CF), neutral detergent fiber (NDF), and acid detergent fiber (ADF) concentrations were determined by a filter bag method (Ankom Technology, Macedon, NY) (Van Soest et al., 1991). The buffering capacity and WSC concentration were measured using the method described by Playne and McDonald (1966) and Murphy (1958), respectively. Hemicellulose concentration was calculated from the difference between NDF and ADF concentrations. Relative feed value (RFV) was calculated based on ADF and NDF (Rohweder et al., 1978).

The pH of the extract was measured using a pH meter (LE438 pH meter, Mettler Toldeo, Shanghai, China). The concentration of organic acid (lactic acid, acetic acid, propionic acid, and butyric acid) was measured by the high performance liquid chromatography (column: Sodex RS Pak KC-811, Showa Denko K.K., Kawasaki, Japan; detector: DAD, 210 nm, SPD-20A, Shimadzu Co., Ltd, Kyoto, Japan; eluent: 3 mmol/L HClO_4_, 1.0 ml/min; temperature: 60°C).

### 2.5 Statistical analysis

The ANOVA method was used to assess the effects and their interactions of years, tillage methods, and maturity stages on the yield, nutritive composition, and silage fermentation quality. When ANOVA indicated a significant F-value (at the 5% probability level), multiple comparisons were performed by Duncan’s multiple range method (SPSS 17.0 for Windows; SPSS Inc., Chicago, IL, USA). The model used was


$$\text{Y}=\mu +{\text{T}}_{i}+\epsilon_{ij},$$

where Y was the response variable, μ was the overall mean, T_i_ was the effect of treatment, and ϵ_ij_ was the residual error.

Relationships among yield and nutritional composition or among nutritional composition and silage fermentation quality of forage were examined by Pearson correlation. Analyses for the yield, chemical composition and silage fermentation quality of different years, tillage methods, and maturity stages were carried out using the principal component analysis function. Based on the information of WCW yield, nutritive composition, and silage fermentation quality, the fuzzy membership function analysis method was used to sort the years, tillage methods, and maturity stages (OriginPro^®^2021b software, OriginLab Corp., WA, USA).

## 3 Results

### 3.1 Yield and agronomic characters

**Table 1** indicates the harvesting time occurred later in growth stage in 2016-2017 than in 2017-2018 and 2018-2019. As shown in **Table 2**, the DM and CP yields were affected by three sources of variation: years, maturity stages, and year × maturity stage interaction (*P*< 0.05). Although tillage methods showed no significant effects on the yield, plant height, and stem number per plant of WCW (*P* > 0.05), compared to CT, the DM and CP yields of NT decreased by 0.74 t/ha and 0.13 t/ha. The DM and CP yields of WCW in 2016-2017 (6.75 t/ha and 0.71 t/ha) were higher than those in 2017-2018 (3.19 t/ha and 0.43 t/ha) and 2018-2019 (2.73 t/ha and 0.34 t/ha) (*P*< 0.05). The WCW at the milk stage had greater DM (5.25 t/ha) and CP (0.60 t/ha) yields than that at the flowering stage (3.19 t/ha and 0.39 t/ha) (*P*< 0.05).

**Table 2:** Yield, plant height and stem number per plant of different years, tillage methods and maturity stages (n=36).

Years and treatments		Dry matter yield (t/ha)	Crude protein yield (t/ha)	Relative feed value	Plant height (cm)	Stem number per plant
Years	2016-2017	6.75±0.74a	0.71±0.10a	107±2.79	81.1±1.37a	1.93±0.23a
	2017-2018	3.19±0.43b	0.43±0.05b	105±2.90	79.7±2.06a	1.67±0.06a
	2018-2019	2.73±0.15b	0.34±0.02b	103±3.28	59.8±1.04b	1.11±0.02b
Tillage methods	CT	4.59±0.62	0.56±0.07	103±2.54	74.0±2.61	1.50±0.08
	NT	3.85±0.55	0.43±0.06	107±2.27	73.1±2.72	1.64±0.18
Maturity stages	Flowering stage	3.19±0.36b	0.39±0.02b	96.3±1.44b	71.4±2.47	1.63±0.18
	Milk stage	5.25±0.70a	0.60±0.08a	114±1.11a	75.7±2.76	1.51±0.08
*P* value	Year (Y)	0.000	0.001	0.533	0.000	0.001
	Tillage method (TM)	0.377	0.159	0.242	0.812	0.451
	Maturity stage (MS)	0.011	0.016	0.000	0.251	0.537
	Y × TM	0.668	0.525	0.838	0.434	0.346
	Y × MS	0.001	0.000	0.298	0.063	0.295
	TM × MS	0.804	0.795	0.370	0.335	0.740
	Y × TM × MS	0.785	0.681	0.224	0.303	0.516

Notes: different lowercase letters in the same column represent significant difference between experiment years, tillage methods or maturity stages (P < 0.05). NT, no-tillage; CT, conventional tillage.

### 3.2 Chemical and microbial compositions

As shown in **Table 3**, the WCW at the milk stage had greater DM, NFE, HC, and WSC concentrations than that at the flowering stage, while the concentrations of CP, CF, ether extract, crude ash, NDF and ADF was lower (*P<* 0.05). Compared with at the milk stage, the WSC concentration at the flowering stage decreased 47.7 g/kg DM, and the NDF and ADF concentrations increased by 41 g/kg DM and 81 g/kg DM, respectively. Therefore, WCW had higher RFV at the milk stage (114) than at the flowering stage (96.3) (*P<* 0.05) (**Table 2**). Tillage methods showed no significant effects on nutritive compositions (*P* > 0.05), but NT tended to reduce NDF and ADF concentrations. The CP and ether extract concentrations in 2017-2018 (140 g/kg DM and 40.9 g/kg DM) and 2018-2019 (126 g/kg DM and 42.1 g/kg DM) were greater than those in 2016-2017 (101 g/kg DM and 22.1 g/kg DM) (*P<* 0.05).

**Table 3:** Nutritional composition of different years, tillage methods and maturity stages (n=36).

Years and treatments		Dry matter (g/kg, FM)	Nutritional composition (g/kg DM)								
			Crude protein	Crude fiber	Ether extract	Crude ash	Nitrogen free extract	Neutral detergent fiber	Acid detergent fiber	Hemicellulose	Water-solublecarbohydrates
Years	2016-2017	246±12.3	101±5.07b	101±5.07	22.1±2.25b	47.4±2.11b	520±15.0a	574±7.90	294±13.5	281±11.0	144±5.14a
	2017-2018	245±14.6	140±6.78a	140±6.78	40.9±1.79a	67.3±3.24a	438±23.4b	594±9.85	285±11.1	309±7.16	115±10.7b
	2018-2019	253±8.65	126±7.56a	126±7.76	42.1±1.70a	46.2±4.01b	480±25.1ab	597±8.88	301±15.5	296±9.19	95.9±10.7b
Tillage methods	CT	245±9.10	126±6.79	318±13.5	32.7±3.01	54.5±3.98	468±21.4	595±7.63	301±11.1	294±6.89	117±9.93
	NT	252±10.4	118±6.26	302±8.86	37.4±2.23	52.8±2.91	490±16.3	582±7.21	286±10.4	297±8.87	120±7.54
Maturity stages	Flowering stage	210±3.73b	132±8.19a	349±8.27a	39.2±2.46a	60.5±3.38a	419±16.0b	609±6.40a	334±5.22a	275±6.25b	94.3±6.77b
	Milk stage	286±2.98a	113±3.18b	270±4.09b	30.9±2.57b	46.8±2.72b	539±7.70a	568±4.87b	253±4.15b	315±6.30a	142±6.49a
*P* value	Year (Y)	0.897	0.001	0.898	0.000	0.000	0.039	0.160	0.722	0.113	0.003
	Tillage method (TM)	0.641	0.367	0.326	0.214	0.739	0.430	0.235	0.316	0.804	0.816
	Maturity stage (MS)	0.000	0.041	0.000	0.027	0.003	0.000	0.000	0.000	0.000	0.000
	Y × TM	0.919	0.751	0.682	0.082	0.292	0.632	0.897	0.782	0.849	0.927
	Y × MS	0.002	0.000	0.769	0.789	0.115	0.002	0.268	0.208	0.295	0.070
	TM × MS	0.306	0.795	0.033	0.953	0.578	0.189	0.372	0.507	0.749	0.482
	Y × TM × MS	0.023	0.276	0.339	0.014	0.179	0.246	0.148	0.999	0.260	0.689

Notes: different lowercase letters in the same column represent significant difference between experiment years, tillage methods or maturity stages (P < 0.05). NT, no-tillage; CT, conventional tillage. FM, fresh matter; DM, dry matter.

The tillage methods had no significant effects on the buffering capacity, pH, and numbers of all microorganisms (*P* > 0.05) (**Table 4**). The pH and numbers of all microorganisms were significantly affected by years (*P*< 0.05). The highest numbers of aerobic bacteria (7.28 lg cfu/g FM) and yeasts (3.48 lg cfu/g FM), and the lowest number of lactic acid bacteria (2.00 lg cfu/g FM) presented on WCW in 2016-2017. The WCW at the milk stage had less aerobic bacteria (6.35 lg cfu/g FM) and yeasts (2.50 lg cfu/g FM) than that at the flowering stage (7.01 lg cfu/g FM and 3.28 lg cfu/g FM) (*P*< 0.05) (**Table 4**).

**Table 4:** Measurements of buffering capacity, pH and microorganism compositions of different years, tillage methods and maturity stages (n=36).

Years and treatments		Buffering capacity (mE kg/DM)	pH	Microorganism compositions (lg cfu/g FM)			
				Aerobic bacteria	Yeasts	Molds	Lactic acid bacteria
Years	2016-2017	163±18.0	6.03±0.04b	7.28±0.21a	3.48±0.16a	5.00±0.23a	2.00±0.19c
	2017-2018	188±4.86	6.08±0.06ab	6.66±0.11b	2.18±0.02b	5.22±0.20a	3.39±0.23a
	2018-2019	197±4.49	6.24±0.07a	6.10±0.16c	3.01±0.27a	3.15±0.13b	2.58±0.14b
Tillage methods	CT	175±10.5	6.15±0.06	6.72±0.21	2.87±0.19	4.40±0.27	2.65±0.21
	NT	190±8.11	6.09±0.04	6.64±0.13	2.91±0.20	4.51±0.28	2.67±0.20
Maturity stages	Flowering stage	172±10.5	6.12±0.06	7.01±0.19a	3.28±0.19a	4.40±0.33	2.88±0.16
	Milk stage	194±7.74	6.11±0.04	6.35±0.11b	2.50±0.14b	4.51±0.21	2.43±0.23
*P* value	Year (Y)	0.090	0.043	0.000	0.000	0.000	0.000
	Tillage method (TM)	0.247	0.447	0.773	0.871	0.773	0.950
	Maturity stage (MS)	0.102	0.942	0.006	0.003	0.788	0.118
	Y × TM	0.018	0.364	0.289	0.763	0.415	0.980
	Y × MS	0.000	0.000	0.032	0.001	0.000	0.110
	TM × MS	0.546	0.943	0.975	0.963	0.880	0.592
	Y × TM × MS	0.147	0.271	0.609	0.940	0.611	0.743

Notes: different lowercase letters in the same column represent significant difference between experiment years, tillage methods or maturity stages (P < 0.05). NT, no-tillage; CT, conventional tillage. FM, fresh matter; DM, dry matter.

### 3.3 Silage fermentation quality

Tillage methods, maturity stages, and their interaction showed no significant effects on the pH value and acetic acid, propionic acid, and butyric acid concentrations of WCW silage (*P* > 0.05) (**Table 5**). Experimental years had significant effects on the pH value, lactic acid, and NH_3_-N concentration of WCW silage (*P*< 0.05). The lactic acid concentration of silage in 2016-2017 (31.6 g/kg DM) were greater than those in 2017-2018 (20.1 g/kg DM) and 2018-2019 (21.4 g/kg DM) (*P*< 0.05), and the lowest NH_3_-N concentration was achieved in 2016-2017 (107 g/kg TN) (*P*< 0.05). The lactic acid concentration of NT (17.1 g/kg DM) was lower than that of CT (26.6 g/kg DM) (*P*< 0.05). WCW silage at milk stage had lower NH_3_-N concentration (125 g/kg TN) than that at flowering stage (169 g/kg TN) (*P*< 0.05) (**Table 5**).

**Table 5:** Silage fermentation measurements of different years, tillage methods and maturity stages (n=36).

Years and treatments		pH	Organic acids (g/kg DM)				NH_3_-N(g/kg TN)
			Lactic acid	Acetic acid	Propionic acid	Butyric acid	
Years	2016-2017	4.54±0.08a	31.6±4.94a	14.6±1.12	21.8±4.15	11.0±2.21	107±14.5b
	2017-2018	4.21±0.05b	12.6±3.25b	20.1±2.24	21.8±3.38	11.5±2.79	171±13.8a
	2018-2019	4.26±0.02b	21.4±1.05b	18.1±1.65	30.1±4.09	8.53±0.56	163±8.53a
Tillage methods	CT	4.29±0.05	26.6±3.98a	16.3±1.49	28.8±4.12	11.7±2.09	162±11.3
	NT	4.39±0.06	17.1±2.04b	18.9±1.45	20.3±1.51	8.96±1.10	131±12.1
Maturity stages	Flowering stage	4.37±0.06	21.5±3.96	16.7±1.08	24.2±3.02	9.27±2.02	169±12.5a
	Milk stage	4.31±0.06	22.2±2.64	18.5±1.80	24.9±3.50	11.4±1.27	125±9.30b
*P* value	Year (Y)	0.001	0.002	0.092	0.235	0.565	0.002
	Tillage method (TM)	0.207	0.042	0.229	0.061	0.254	0.068
	Maturity stage (MS)	0.525	0.892	0.378	0.887	0.375	0.007
	Y × TM	0.516	0.088	0.056	0.494	0.968	0.368
	Y × MS	0.512	0.049	0.073	0.172	0.025	0.004
	TM × MS	0.785	0.749	0.229	0.634	0.202	0.643
	Y × TM × MS	0.636	0.423	0.112	0.401	0.203	0.396

Notes: different lowercase letters in the same column represent significant difference between experiment years, tillage methods and maturity stages (*P* < 0.05). NT, no-tillage; CT, conventional tillage. DM, dry matter.

### 3.4 Interrelation pattern among the yield, nutritive composition, and silage fermentation quality

Correlation analyses showed that the DM yield of WCW was negatively correlated with CP, ether extract, crude ash and NDF concentrations (*P*< 0.05) (**Table 6**). The number of aerobic bacteria was significantly positively correlated with the pH of WCW silage (*P*< 0.05). The concentrations of DM and WSC and number of yeasts and lactic acid bacteria of WCW prior to ensiling had a negative correlation to NH_3_-N concentration of WCW silage, while the CP, crude fiber, ether extract, crude ash, ADF, and NDF concentrations of WCW, before ensiling, had a positive correlation to the NH_3_-N concentration of WCW silage (*P*< 0.05). The buffering capacity of WCW before ensiling was negatively correlated with the lactic acid concentration of WCW silage (*P*< 0.01) (**Table 6**).

**Table 6:** Correlation plot of Pearson among yield, nutritional composition and silage fermentation quality of whole crop wheat (n=36).

Items	DMY	CPY	PH	SNPP	DM	CP	CF	EE	CA	NDF	ADF	WSC	BC	pH of FM	AB	Yeasts	Molds	LAB	pH of silage	LA	AA	PA	BA	NH_3_-N
DMY	1.000																							
CPY	0.947^**^	1.000																						
PH	0.516^**^	0.468^**^	1.000																					
SNPP	0.268	0.151	0.484^**^	1.000																				
DM	0.373^*^	0.349^*^	0.120	-0.050	1.000																			
CP	-0.368^*^	-0.069	-0.242	-0.346^*^	-0.336^*^	1.000																		
CF	-0.253	-0.211	-0.088	0.216	-0.769^**^	0.323	1.000																	
EE	-0.834^**^	-0.717^**^	-0.566^**^	-0.297	-0.319	0.570^**^	0.243	1.000																
CA	-0.341^*^	-0.186	0.124	0.044	-0.522^**^	0.638^**^	0.593^**^	0.444^**^	1.000															
NDF	-0.436^**^	-0.337^*^	-0.325	-0.161	-0.605^**^	0.511^**^	0.650^**^	0.460^**^	0.503^**^	1.000														
ADF	-0.311	-0.284	-0.248	0.066	-0.821^**^	0.291	0.827^**^	0.238	0.373^*^	0.688^**^	1.000													
WSC	0.616^**^	0.498^**^	0.581^**^	0.321	0.586^**^	-0.572^**^	-.0575^**^	-.0657^**^	-0.419^*^	-0.647^**^	-0.604^**^	1.000												
BC	-0.056	0.096	-0.321	-0.249	0.327	0.453^**^	-0.264	0.344^*^	0.046	0.075	-0.249	-0.185	1.000											
pH of FM	-0.066	0.106	-0.321	-0.242	0.114	0.471^**^	0.015	0.263	0.007	0.159	0.120	-0.142	0.427^**^	1.000										
AB	0.194	0.086	0.479^**^	0.461^**^	-0.523^**^	-0.247	0.369^*^	-0.414^*^	0.127	0.056	0.406^*^	0.126	-0.622^**^	-0.421^*^	1.000									
Yeasts	0.172	0.068	-0.074	0.203	-0.386^*^	-0.227	0.390^*^	-0.258	-0.207	0.135	0.511^**^	-0.134	-0.383^*^	0.163	0.471^**^	1.000								
Molds	0.156	0.052	0.773^**^	0.543^**^	-0.029	-0.311	-0.058	-0.309	0.215	-0.248	-0.173	0.470^**^	-0.417^*^	-0.486^**^	0.548^**^	-0.153	1.000							
LAB	-0.618^**^	-0.536^**^	-0.068	0.073	-0.320	0.359^*^	0.287	0.550^**^	0.559^**^	0.162	0.174	-0.392^*^	-0.074	-0.213	-0.072	-0.414^*^	0.211	1.000						
pH of silage	0.389^*^	0.324	0.170	-0.019	-0.133	-0.179	-0.126	-0.383^*^	-0.193	-0.080	0.042	0.167	-0.021	-0.136	0.347^*^	0.366^*^	0.127	-0.413^*^	1.000					
LA	0.309	0.176	0.055	0.172	-0.045	-0.485^**^	0.175	-0.485^**^	-0.204	-0.167	0.105	0.270	-0.533^**^	-0.070	0.297	0.446^**^	0.034	-0.279	0.015	1.000				
AA	-0.350^*^	-0.406^*^	0.086	-0.155	0.128	-0.093	-0.196	0.163	-0.095	-0.071	-0.224	0.097	-0.023	-0.212	-0.149	-0.465^**^	0.139	0.205	-0.245	-0.330^*^	1.000			
PA	-0.046	0.006	-0.235	-0.269	0.000	0.171	0.034	-0.006	-0.159	0.080	-0.015	-0.122	0.032	0.171	-0.316	-0.118	-0.284	0.035	-0.239	-0.005	0.380^*^	1.000		
BA	0.232	0.315	0.214	-0.087	0.156	0.172	0.079	-0.275	0.157	0.198	-0.087	0.103	0.228	0.112	-0.071	-0.173	0.093	-0.217	0.014	-0.114	0.207	0.419^*^	1.000	
NH_3_-N	-0.438^**^	-0.241	-0.352^*^	-0.303	-0.441^**^	0.706^**^	0.480^**^	0.490^**^	0.484^**^	0.515^**^	0.390^*^	-0.648^**^	0.046	0.188	-0.140	-0.188	-0.356^*^	0.439^**^	-0.348^*^	-0.246	0.165	0.509^**^	0.188	1.000

Note: Asterisks indicate significant differences at *P* < 0.05 (*) and *P* < 0.01 (**), respectively. DMY, dry matter yield; CPY, crude protein yield; PH, plant height; SNPP, stem number per plant; DM, dry matter; CP, crude protein; CF, crude fiber; EE, ether extract; CA, crude ash; NDF, neutral detergent fiber; ADF, acid detergent fiber; WSC, water-soluble carbohydrates; BC, buffering capacity; AB, aerobic bacteria; LAB, lactic acid bacteria; LA, lactic acid; AA, acetic acid; PA, propionic acid; BA, butyric acid.

**Figure 2:**
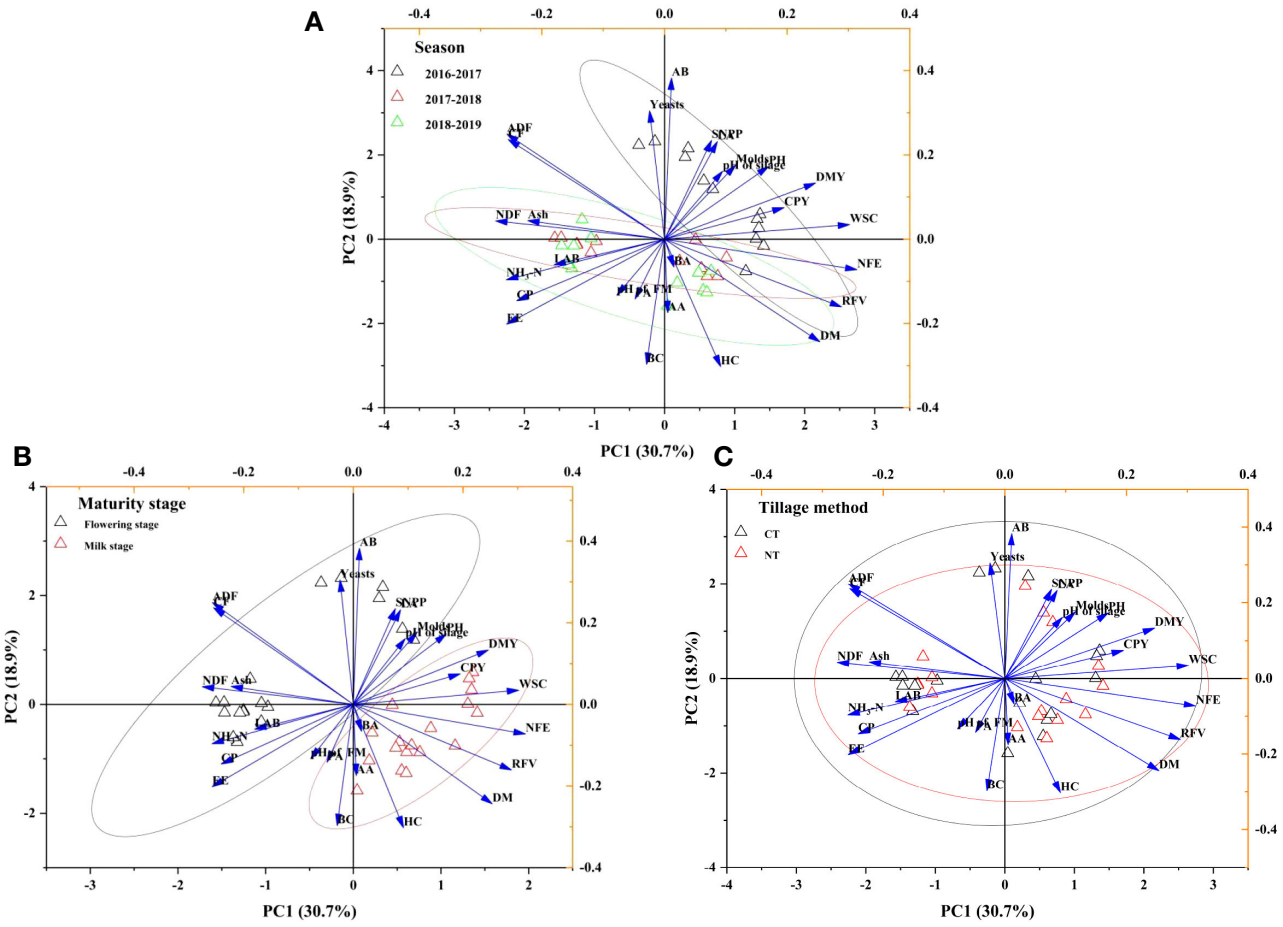
Scatter diagram of principal component analysis of agronomic characts, yields, and relative feed value of different years **(A)**, tillage methods **(B)** and maturity stages **(C)**. The ellipse indicates 95% confidence. NT, no-tillage; CT, conventional tillage. DMY, dry matter yield; CPY, crude protein yield; RFV, relative feed value; PH, plant height; SNPP, stem number per plant; FM, fresh matter; DM, dry matter; CP, crude protein; CF, crude fiber; Ash, crude ash; EE, ether extract; NFE, nitrogen free extract; NDF, neutral detergent fiber; ADF, acid detergent fiber; HC, hemicellulose; BC, buffering capacity; AB, aerobic bacteria; LAB, lactic acid bacteria; LA, lactic acid; AA, acetic acid; PA, propionic acid; BA, butyric acid.

The interrelationships of yields and quality indicators of WCW were relatively consistent and steady regardless of tillage methods, while silage fermentation quality indicators varied between maturity stages (**Tables 2**, **3**, **5**; **Figure 2**). Twenty-seven initial variables were reduced to four variables that contained information about most of the original variables (four indicators were not related to each other) (not listed). The eigenvalues of these four principal components were all greater than 1, indicating the principle of extraction as principal components was reasonable. In the initial eigenvalues, the cumulative variance contribution rate of the four principal components extracted and loaded was 96.5%. Specifically, the WCW at the milk stage had the highest yield, DM, and WSC content (**Tables 2**, **3**), which was clearly separated from the flowering stage along the first axis (**Figure 2**). On the other hand, 2016-2017 had the highest yield, WSC and NFE contents, which was clearly separated from the other two years along the first axis (**Figure 2**). However, there were no differences in most yield, nutrients, and silage fermentation quality of two tillage methods. This was also the reason that the 95% confidence intervals overlap. According to the results of the membership function analysis, 2017-2018 scored the highest in years, NT scored the highest in tillage methods, milk stage scored the highest in maturity stages (**Supplementary Table 1**).

## 4 Discussion

### 4.1 Agronomic characters and yields

The DM and CP yields in 2016-2017 were greater than those in 2017-2018 and 2018-2019 (*P*< 0.05). The growth time of WCW in 2016-2017 was longer than that in 2017-2018 and 2018-2019 in both flowering and milk stages (**Table 1**), which was conducive to the accumulation of more photosynthetic products and increased the yield of WCW. Additionally, in Nov. and Dec. of 2016-2017, there was higher rainfall (64 mm and 14 mm) combined with higher temperatures (19.6°C and 16.7°C), which was beneficial for the accumulation of biomass in wheat early-stage. Several studies have found that climate has a greater impact on the yield and nutritive composition of forage crops (Ergon et al., 2018; Druille et al., 2020). However, the differences in temperature and rainfall between two years in this study, and wheat had similar morphological and anatomical at harvest (2017-2018 and 2018-2019). Principal component analysis also demonstrated a high overlap between 2017-2018 and 2018-2019 wheat in terms of yield, nutrient content and information on silage fermentation quality (**Figure 2A**).

Notably, moisture and temperature were the two most important factors affecting the growth process of crops. In this study, due to the perennial planting of early and late rice in the region, the shallow soil water distribution, which had a positive effect on the growth of the winter crop (low rainfall in winter). In addition, the sufficient light and heat were available for crop growth in winter. The above factors were all conducive to the growth of wheat. To pursue a higher DM yield, WCW was harvested after the dough stage (Filya, 2003). However, this is not allowed when the crop rotation system is more complex, as it might cause a delay in the sowing time of the subsequent crops, which in turn reduces the economic profitability of the rotation system. It was noted that the yield of wheat in this study was lower than those of some published data (Filya, 2003; Xie et al., 2012). Except for genotype, the reasons for these results were mainly related to the different growth stage and planting environment of wheat.

In the previous study, we found that the population density of wheat by NT (1.54 × 10^6^ plant/ha) was significantly lower than that of CT (2.09 × 10^6^ plant/ha), and similar results were obtained in the experiment of no-tillage Italian ryegrass (*Lolium multiflorum*) (Xu et al., 2021a). However, there were no differences in the DM and CP yields between NT and CT in this study. Furthermore, there were no differences in plant height and stem number per plant between NT and CT (**Table 2**). This shows that the per plant dry matter and crude protein accumulation rates of NT were higher than that of CT, which can explain the consistency of their yields. Light interception has been proved to increase with the increase of population density, while the effective radiation of photosynthesis penetrating the lower vegetation layer decreases (Yang et al., 2019). This also explains that the stem number per plant of NT was slightly more than that of CT (**Table 2**). Except for climatic factors, soil nutrients and moisture were considered to be the main factors causing variation in the yield of crop, and proper soil structure was essential to improve soil fertility and water concentration (Xu et al., 2021b). A global meta-analysis showed that no differences in the yield of crops (rice, maize, barley, and soybean) were observed between NT and CT (Shakoor et al., 2021). In the previous study, we found that no-tillage reduced disturbance and preserved more nutrients and water in soil than CT (Xu et al., 2021b). Furthermore, under NT conditions, soil water evaporation was limited and more water was available to the crop. In this study, the key factor affecting the growth of WCW was rainfall in different years. Therefore, we speculate that NT treatment suppressed the evaporation of soil water and ensured that wheat had an adequate water supply during the filling period. It was worth noting that there were differences in water supply between tillage treatments (Xu et al., 2021b). This also compensated for the risk of yield reduction due to lower population density. The reason maybe that the seeds were directly sown on the surface of the soil, resulting in a low germination rate in this study (small contact area between seed and soil, weak water absorption of seed, and no irrigation). The sample size (3 plots replicates of 1 × 1 m^2^) was supposed to alter the results. Principal component analysis also demonstrated a high overlap between NT and CT wheat in terms of yield, nutrient content and information on silage fermentation quality (**Figure 2C**). However, the results based on the analysis of the affiliation function showed that the years and maturity stages had a great impact on forage yield and nutrition (**Supplementary Table 1**).

The greater DM and CP yields at the milk stage than at the flowering stage were due to the longer development cycle of WCW (**Tables 1**, **2**), accumulating greater photosynthetic products. This was consistent with the results of some studies about delayed grass harvest (Xie et al., 2012). Compared to flowering stage, the DM and CP yields at the milk stage increased by 1.35 t/ha and 0.21 t/ha, respectively. Evidently, WCW accumulated more photosynthetic products at milk stage.

### 4.2 Nutritive and microbial compositions

The WCW at milk stage had lowter NDF and ADF concentrations than at flowering stage (*P<* 0.05). Many research results showed that high concentrations of NDF and ADF had a negative impact on forage digestive effectiveness (Monteiro et al., 2021). The population density of CT was high, intensifying the competition for nutrients among plants. Several studies had also provided similar conclusions (Obour et al., 2019). It was worth noting that tillage methods showed no significant effects on the nutritive composition of WCW (*P* > 0.05). The sowing methods does not affect the nutrition of WCW through density and soil characteristics. It suggests that sowing methods does not affect nutritive composition of WCW through population density and soil properties. Similar results were obtained in the experiments of maize (Wang et al., 2020) and soybean (Yusuf et al., 1999). With increasing maturity, the ratio of grains to whole plants increased. This explains the higher DM concentration of WCW at flowering stage.

Previous studies have shown that the growth stage (or harvest time) was an important factor affecting the chemical composition of grasses (Stirling et al., 2022). In the early stages of growth, the leaves have a high proportion of the whole plant in grasses. Therefore, grasses have a high CP concentration. In this study, the NDF and ADF concentrations of WCW decreased by 6.7% and 24.3% from the flowering to the milk stage, respectively, whereas WSC concentration increased by 50.6%. Similar trends had been observed in some studies (Xie et al., 2012). WCW at the milk stage had lower CP concentration than that at the flowering stage (*P*< 0.05). The reason was that protein synthesis was weakened (dilution effect) as the growth period was delayed. Conversely, starch accumulation offsets lower NDF digestibility. Unfortunately, we did not study the WCW at the dough stage.

Before ensiling, there were many kinds of microorganisms on the surface of grasses, which would produce different metabolites during the ensiling and in turn determine the fermentation quality of silage. The number of aerobic bacteria was much higher than those of other microorganisms in this study. The number of lactic acid bacteria was not affected by the maturity stages (**Table 4**).

### 4.3 Silage fermentation quality

Ensiling was an effective way to preserve the nutritional quality of grasses and was a global practice to preserve the moist forage crops when drying forage is difficult (especially in the rainy season). Moreover, the general practice was to wilt the crop before ensiling, if water content is high at harvest. Years had a large influence on the silage fermentation quality of WCW in this study, while the effects of tillage methods and maturity stages on the silage fermentation quality of WCW were very limited (**Table 5**). This was a key issue because population density was related to the epiphytic microbial population (**Table 1**) and could also affect the fermentation profile. In this study, WCW at the milk stage had a lower NH_3_-N concentration than that at the flowering stage. This was because the higher WSC concentration of WCW at the milk stage provided more substrate for the rapid propagation of lactic acid bacteria. Moreover, the rapid reduction of pH in anaerobic environment inhibited plant and microbial protease activities, subsequently inhibiting protein degradation. Several studies had also provided similar conclusions (Xie et al., 2012). Thus, WSC concentration was negatively correlated to NH_3_-N concentration (**Table 6**), and it was adopted as a positive indicator in the evaluation of forage feeding potential. Although we did not study the microbial species in silage, we speculations there was substantial evidences of clostridial activity throughout. Across all silages, butyric acid content was >1.0% DM, while NH_3_-N accounted for roughly 15% of total N. Also, the propionic acid concentration was very high (about 2.5%) in all the treatments. All of these responses indicate significant clostridial activity.

## 5 Conclusions

This study shows that years had a strong influence on the yield and nutritional composition of whole-crop wheat in southern China. This was mainly related to the amount of rainfall, as it affects the seedling emergence rate of wheat. In addition, the interrelation patterns among the yields (DM and CP), nutritional compositions and silage fermentation quality indicators were not affected by tillage methods. Wheat sown by NT and CT was of similar yield and nutritional value, irrespective of harvest stages. WCW harvested at the milk stage had greater yield and better nutritional composition and silage fermentation quality than that at the flowering stage. No-tillage sowing of wheat was feasible and harvesting at the milk stage was recommended.

## Data availability statement

The original contributions presented in the study are included in the article/**Supplementary Material**. Further inquiries can be directed to the corresponding author.

## Author contributions

LX: Data curation, Investigation, Writing – original draft, Writing – review & editing. GT: Investigation, Writing – review & editing. DW: Data curation, Investigation, Writing – review & editing. YH: Data curation, Investigation, Writing – review & editing. JZ: Data curation, Funding acquisition, Investigation, Methodology, Writing – review & editing.
