# Supplementary material for: Effects of tillage and maturity stage on the yield, nutritive composition, and silage fermentation quality of whole-crop wheat
Source: Front Plant Sci. 2024 Mar 27;15:1357442. doi: 10.3389/fpls.2024.1357442 (PMC11008282; doi:10.3389/fpls.2024.1357442)
Supplement: Supplementary file 1 [file Table_1.docx]

**Table S1** Scores and rankings of different years, tillage methods and maturity stages

| Season and treatments | | Factor analysis value (FAC, extracting the sum of squared loads) | | | | Composite scores | Ranking in composite scores |
| --- | --- | --- | --- | --- | --- | --- | --- |
|  |  | FAC 1 | FAC 2 | FAC3 | FAC4 |  |  |
| Years | 2016-2017 | 0.72369 | -1.60122 | 0.96313 | 0.0622 | 0.5446 | 2 |
|  | 2017-2018 | -0.62061 | 1.53706 | 0.88964 | 0.64335 | 0.7864 | 1 |
|  | 2018-2019 | 0.00097 | 0.08225 | -1.86709 | -0.73365 | 0.0525 | 3 |
| Tillage methods | CT | -0.46243 | -0.48493 | -0.59415 | 1.50343 | 0.094 | 2 |
|  | NT | 0.41428 | 0.47162 | 0.59039 | -1.48154 | 0.7733 | 1 |
| Maturity stages | Flowering stage | -1.56159 | -0.5543 | 0.22483 | -0.53944 | -0.3823 | 2 |
|  | Milk stage | 1.50569 | 0.54952 | -0.20676 | 0.54565 | 1.2517 | 1 |

Note: The variance contribution rate of principal component analysis 1, principal component analysis 2, principal component analysis 3, and principal component analysis 4 were 44.4%, 26.0%, 19.7%, and 6.44%, respectively, and initial eigenvalue > 1.
